# Comparing Exciton–Plasmon Couplings in J‐Aggregate and TMD‐Based Hybrids Using Angle‐Resolved Two‐Dimensional Electronic Spectroscopy

**DOI:** 10.1002/nap2.70088

**Published:** 2026-04-17

**Authors:** Daniel C. Lünemann, Daniel Timmer, Antonietta De Sio, Christoph Lienau

**Affiliations:** ^1^ Institut für Physik, Carl von Ossietzky Universität Oldenburg Germany; ^2^ Center for Nanoscale Dynamics (CeNaD) Carl von Ossietzky Universität Oldenburg Germany

**Keywords:** exciton–plasmon coupling, strong coupling, two‐dimensional electronic spectroscopy, ultrafast spectroscopy

## Abstract

Recent developments in ultrafast two‐dimensional electronic spectroscopy (2DES), a powerful tool to unravel coherent couplings in nanosystems, have enabled studies of strongly coupled collective excitons and surface plasmon polaritons. Here, we report a comparative study of two prototypical systems, plasmonic nanoslit arrays covered with either a J‐aggregated molecular thin film or a transition metal dichalcogenide (TMD) monolayer. Using ultrafast angle‐resolved 2DES, we probe the coherent polariton response. We show that in both systems dark or weakly coupled excitons play an essential role for the polariton nonlinearity. In J‐aggregate‐based hybrids, this results in dominant Rabi oscillations with periods differing from the polariton normal mode splitting. In TMD‐based hybrids, we observe a largely enhanced polariton nonlinearity and polariton dynamics showing a rapid relaxation into dark states. We rationalize the observations in both systems using phenomenological model Hamiltonian simulations and show that the microscopic origin of the polariton nonlinearities in the two systems are different. Although the characteristic two‐exciton blue‐shift is dominant in the J‐aggregate‐based hybrid, many‐body interactions result in a pump‐induced reduction in normal mode splitting in the TMD‐based system. We anticipate that the presented results will be helpful for the design of active exciton–plasmon systems for ultrafast switching and optical information processing.

## Graphical abstract

Polaritons—hybrid light‐matter states—allow for flexibly tailoring the optical response of materials. The authors compare ultrafast, two‐dimensional electronic spectroscopy studies of different classes of polaritons, formed by coupling a plasmonic grating to either excitons in a thin film of molecular J‐aggregates or a WS2 monolayer. Different microscopic optical nonlinearities, relevant dark states and potential for ultrafast switching are demonstrated.

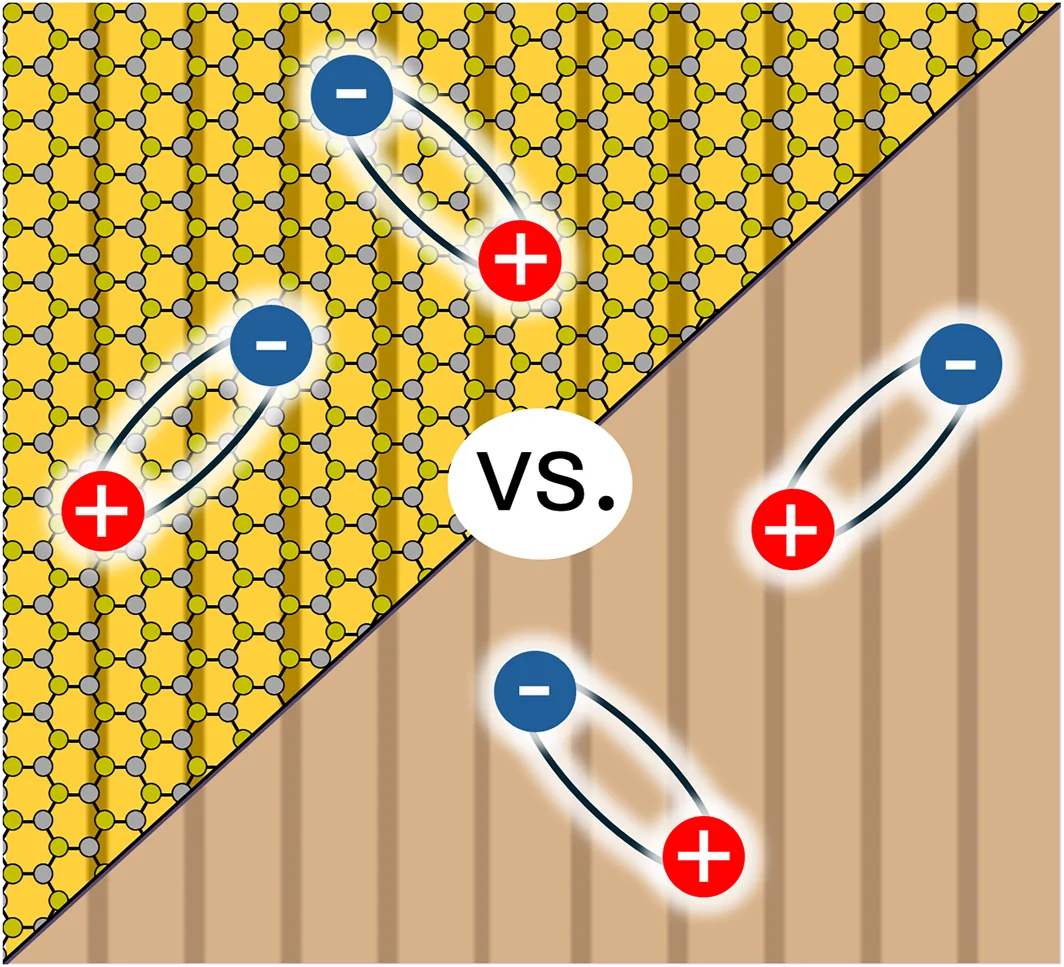

## 1 Introduction

The strong coupling of molecular and semiconducting quantum emitters to plasmonic and dielectric cavity modes has raised renewed interest over the last few years [1, 2, 3, 4, 5, 6, 7, 8, 9, 10, 11]. This interest is partially triggered by the prospect to tailor optical and electronic properties of the quantum emitter materials via their coupling to fluctuating electromagnetic fields [6, 7, 8, 9, 10, 11, 12, 13, 14, 15]. Moreover, new ideas emerge how to exploit such coupling for an efficient ultrafast switching of light by light on the nanoscale [6, 16] and for the development of novel active metamaterials [9, 10, 11, 12, 13, 17, 18, 19].

Another reason for this growing interest are rapid developments in the experimental implementation of two‐dimensional electronic spectroscopy (2DES) [20, 21, 22, 23, 24]. Using tailor‐designed pulse sequences for the sample excitation, 2DES is ideally suited for unraveling coherent dynamics, couplings, and energy flow in hybridized systems [4, 5, 23, 24, 25, 26]. As such, experimental activities in probing strong couplings using 2DES are growing quickly [4, 5, 9, 23, 27, 28, 29]. At the same time, a substantial body of theoretical work has investigated how such strong couplings affect time‐dependent 2DES spectra [1, 2, 3, 30, 31, 32, 33, 34, 35, 36].

Here, we discuss how the collective coupling of two largely different and complementary classes of quantum emitters, specifically J‐aggregated molecular excitons [37, 38] and excitons in atomically thin semiconductors [39], to nanostructured electromagnetic fields in plasmonic cavities [4, 5], manifest in two‐dimensional electronic spectroscopy. The optical properties of these two classes of quantum emitters are vastly different. J‐aggregated molecular excitons are spatially highly localized Frenkel‐type excitations with a large optical transition dipole moment, resulting in typical thin‐film absorptions close to unity [37, 38]. For monolayers of transition metal dichalcogenides (TMD), the absorption is much reduced, despite the large transition dipole moment of their Wannier‐type excitons [39]. Additionally, the physical mechanisms underlying the optical nonlinearities of these quantum emitters are largely different. For J‐aggregated excitons, the combination of quantum confinement and Pauli blocking results in a characteristic few‐meV blue shift of their two‐exciton excitations [22, 37]. For TMDs, excitonic nonlinearities mainly arise from many‐body interactions, specifically excitation induced dephasing [5, 39, 40, 41].

We explore the effects of these different optical properties of these quantum emitters on the 2DES spectra of hybrid nanostructures comprising a prototypical plasmonic nanoresonator, a metal nanoslit array covered with either a thin J‐aggregate squaraine layer [4] or a monolayer (1L) of tungsten disulfide (WS_2_) [5]. We exploit angle‐resolved 2DES with 10‐fs time resolution to investigate the effects of exciton–surface plasmon polariton formation on the 2DES spectra. The high time resolution gives access to the coherent exciton–plasmon coupling dynamics, a coherent energy transfer between plasmons and excitons, which underlies the formation of the polariton modes. In both systems, the subwavelength in‐plane confinement of the structured plasmonic field induces pertinent couplings between optically dark and bright excitons that go beyond the typical two‐mode description of strong coupling phenomena [42]. The difference in transition dipole moment and damping of the two types of quantum emitters has profound effects on the 2DES spectra of the coupled system. For superradiant J‐aggregates, the nonlinearity of one of the polaritonic modes becomes vanishingly small, whereas the optical nonlinearities of TMD monolayers are strongly enhanced by the coupling to the plasmons. Our experiments reveal both phenomena and their effects on the 2DES spectra. They are understood in the framework of phenomenological open‐system master equations that account for decoherence and many‐body effects. As such, our results open a path toward the design of strongly coupled quantum systems and artificial metamaterials with possible applications in ultrafast and efficient optical information processing.

## 2 Results and Discussion

We fabricated two hybrid exciton/plasmon nanostructures. For the J‐aggregate/plasmon hybrid sample, a planar polycrystalline gold film with a thickness of 200 nm is deposited on a fused silica substrate and thermally annealed. A nanoslit array made of 45 nm wide and deep grooves with a period of 530 nm is milled into the surface using a focused gallium ion beam. The whole nanoslit array is covered with a ∼10‐nm‐thick layer of the J‐aggregated squaraine dye ProSQ‐C16 [37, 43]. After spin‐coating with a ProSQ‐C16 solution, the film is thermally annealed to improve the homogeneity of the aggregate layer [4, 37]. The resulting hybrid sample is sketched in Figure 1a. Whereas the molecular J‐aggregate resonance energy $E_{X}$ is independent of the angle of incidence θ, the surface plasmon polariton (SPP) resonance energy $E_{\text{SPP}}$ of the grating can be tuned using θ. Figure 1b shows characteristic reflectivity spectra of the nanoslit array without J‐aggregate layer (black line) and of the J‐aggregate layer on a planar region of the gold film (red line). A characteristic spectrum of the hybrid system is shown as a blue line. The spectra of the nanoslit array and of the hybrid sample have been recorded with p‐polarized light (perpendicular to the nanoslits) at an angle of θ=25°, indicated by a black dashed line in Figure 1c. The finite background dielectric function of the J‐aggregate layer results in a red‐shift of the SPP resonances of the array with respect to those of the bare array. In the hybrid sample strong dipolar couplings between the SPP and the ensemble of molecular excitons results in the formation of collective upper (UP) and lower (LP) polaritons. The strong LP resonance appears on the red side of the spectrum, whereas, at this incidence angle, the weaker UP resonance overlaps with a pronounced angle‐independent peak at the main exciton resonance of the bare J‐aggregate film. This angle‐independent resonance has initially been assigned to excitons in regions outside of the nanoslits with weak couplings to the SPP modes of the array [27]. Simulations of Maxwell's equations (Lumerical) [4] confirm that, in the absence of the plasmonic field, these excitons should be considered as Frenkel‐type excitons that are localized in regions outside of the slits. Their coupling to largely detuned higher‐order grating modes should not be neglected since these couplings may increase the localization length of their center‐of‐mass wave function [37]. The results presented below show that these “uncoupled” excitons also exhibit a finite degree of hybridization with the SPP mode and excitons in the slit region [4]. The dependence of the hybrid resonances on the incidence angle is seen in Figure 1c. These spectra now reveal the UP resonance for θ<25°, in addition to the LP and “uncoupled” exciton (X) resonances. An avoided crossing between UP and LP at θ∼23° is a clear indication for strong X‐SPP coupling. For larger angles, the detuning $\Delta =E_{\text{SPP}}-E_{X}$ is negative, while for smaller angles Δ becomes positive. An analysis of the spectra reveals a normal mode splitting (NMS) of ∼60 meV [4]. As a consequence of the X‐SPP coupling, the transition dipole moments of LP and UP are given as in‐phase (out‐of‐phase) superpositions of the effective transition dipole moments of the exciton ensemble and of the plasmonic mode. In the J‐aggregate hybrid, these dipole moments are comparable in strength [44]. Hence, their destructive interference results in a suppression of the UP resonance close to the crossing angle while the LP resonance gains oscillator strength.

**FIGURE 1:**
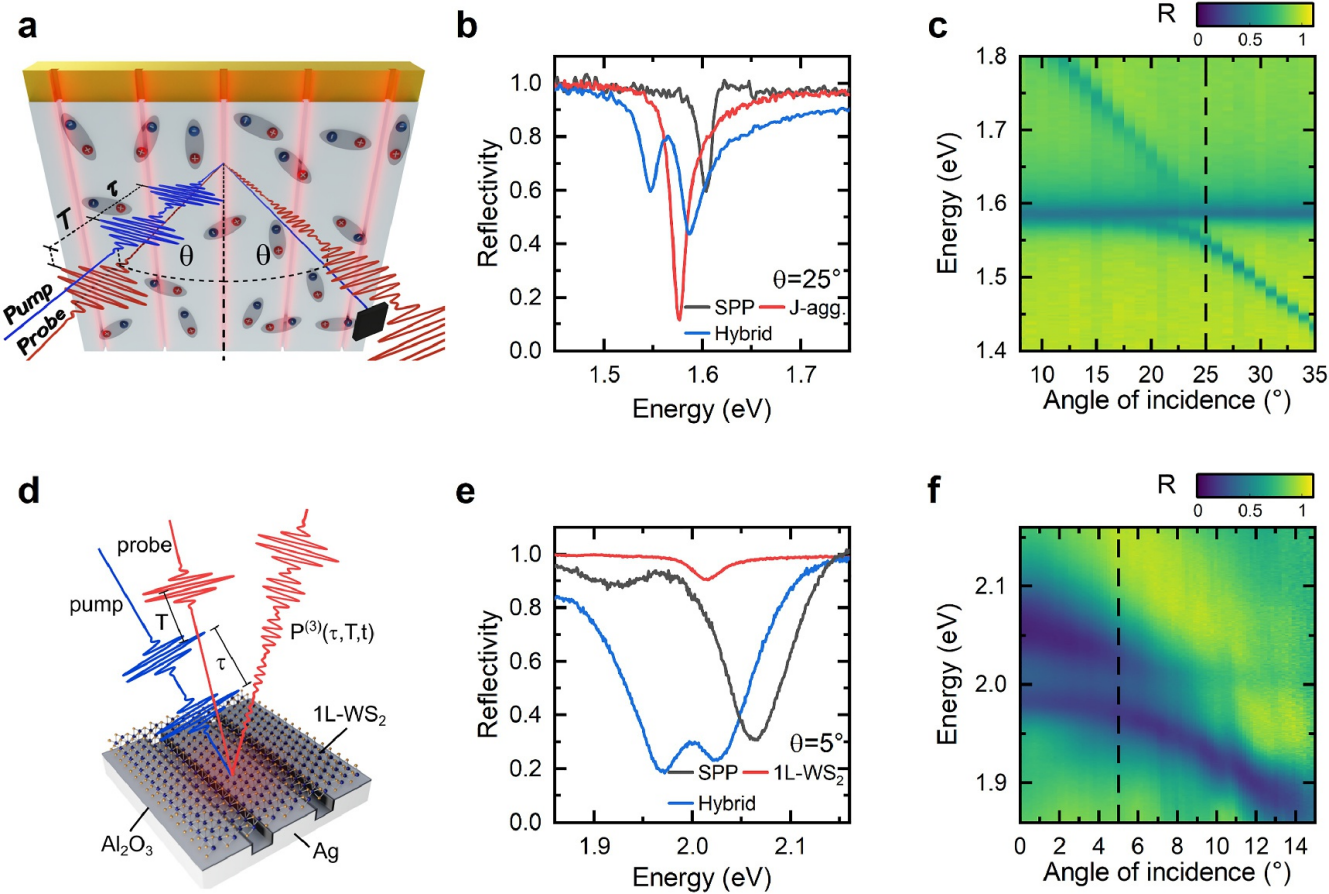
Two‐dimensional electronic spectroscopy (2DES) of hybrid exciton/plasmon nanostructures. (a) Experimental geometry of the molecular J‐aggregate sample. A gold nanoslit array with 530‐nm period is covered with a 10‐nm thin film of J‐aggregated ProSQ‐C16 molecules. A phase‐locked pair of pump pulses with delay τ (blue) and a probe pulse, delayed by the waiting time T, are focused onto the sample at incidence angle θ. The reflected probe light is detected. (b) Reflection spectrum of the bare nano‐slit array (black), the J‐aggregated thin‐film on an unstructured Au surface (red) and the strongly‐coupled hybrid, J‐aggregate/plasmon system (blue) at an angle of θ=25°. (c) Angle‐resolved reflectivity spectra of the hybrid system reveal the dispersion relation of upper (UP) and lower (LP) polaritons and an angle‐independent peak at the bare exciton resonance. (d) Experimental geometry of the hybrid 1L‐WS_2_ on silver sample. A silver nanoslit array with 495‐nm period is coated with 5 nm Al_2_O_3_ and covered with a monolayer of WS_2_. The same experimental geometry as in (a) is used. (e) Reflection spectrum of the bare nano‐slit array (black), the 1L‐WS_2_ layer on a planar silver surface (red) and the coupled 1L‐WS_2_/plasmon system (blue) at an angle of θ=5°. In the presence of the monolayer, the surface plasmon polariton resonance red‐shifts by ∼90 meV. (f) Angle‐resolved reflectivity spectra of the coupled 1L‐WS_2_/plasmon system reveal the UP and LP dispersion relations.

For comparing strong X‐SPP coupling dynamics in J‐aggregate/plasmon hybrids to those in TMD‐based samples, we fabricated a sample consisting of a similar nanoslit array, now covered with a monolayer of WS_2_. The array is formed in a polycrystalline silver film by engraving a nanoslit array with a period of 495 nm. The slits have a width and depth of 45 nm and are also formed using focused gallium ion beam milling [5]. To avoid electron transfer between TMD layer and silver, a 5‐nm Al_2_O_3_ layer is coated onto the silver surface. A WS_2_ monolayer, exfoliated from 2H‐WS_2_, is dry‐transferred onto the nanoslit array [5, 40]. The resulting structure is shown in Figure 1d. Figure 1e shows the SPP resonance of the bare silver array, before adding the 1L‐WS_2_ layer, as a black line. When deposited on a planar silver film, the 1L‐WS_2_ layer shows a weak dip in reflectivity as signature of its A‐exciton resonance (red line). A selected spectrum of the hybrid sample, recorded with p‐polarized light at an angle of θ=5°, is depicted as a blue line in Figure 1e. Again, the SPP resonance of the bare array is blue‐shifted with respect to that of the 1L‐WS_2_/plasmon sample. In the hybrid sample, X‐SPP coupling leads to two resonances with almost equal strength, which we associate with the newly formed UP and LP states with a NMS of 60 meV [5]. Because the A‐exciton transition dipole moment of 1L‐WS_2_ is much weaker than the effective dipole moment of the SPP modes, the oscillator strengths of the UP and LP states near the crossing are comparably large and essentially given by that of the SPP resonance. Angle‐dependent reflectivity spectra in Figure 1f again reveal an avoided crossing between UP and LP, now at θ≈6°. Therefore, the detuning in this sample is negative for larger θ and positive for smaller θ. The spectra show that the sample is near the onset of the strong coupling regime with a NMS that slightly exceeds the average dephasing rates of the uncoupled modes [5].

Figure 1a,d show the experimental geometry for the 2DES measurements. A phase‐locked pair of two collinear pump pulses [45] with essentially identical time structure and with a relative delay of τ, the coherence time, is focused onto the sample under an incidence angle of θ (blue). A probe pulse (red), delayed by the waiting time T with respect to the second pump pulse, is also focused onto the sample under a small angle with respect to the pump. All beams are linearly p‐polarized perpendicular to the nanoslits. The 2DES signal that is emitted in the direction of the reflected probe beam is collected [22, 46]. We record the probe spectrum in the presence ($I_{\text{on}}$) and absence ($I_{\text{off}}$) of the pump‐pulses as a function of the interpulse delays τ and T. Differential reflectivity spectra are then calculated as the pump‐induced relative change of the probe spectrum, $\Delta R/R=\left[I_{\text{on}}\left(\tau ,T,E_{\det}\right)-I_{\text{off}}\left(E_{\det}\right)\right]/I_{\text{off}}\left(E_{\det}\right)$. Finally, Fourier transform of the ΔR/R spectra along τ at a given T yield absorptive two‐dimensional electronic spectra $A_{2D}\left(E_{\text{ex}},T,E_{\det}\right)$ as a function of excitation ($E_{\text{ex}}$) and detection ($E_{\det})$ energy for each waiting time [22, 46, 47]. Because of the reflecting sample substrate, these reflectivity spectra are effectively recorded in a double‐transmission and are thus showing absorptive lineshapes [37, 40, 48].

Figure 2a shows the 2DES map of the bare J‐aggregated thin film at T=0, revealing a single, dispersive feature along the detection axis. This dispersive lineshape is a characteristic signature of a J‐aggregated Frenkel exciton system in which Pauli blocking within each monomer results in a finite blue‐shift of the two‐exciton states in the aggregate [49, 50]. This gives rise to a blue‐shift of their excited state absorption (ESA) with respect to the exciton transition [49]. In our ΔR/R and absorptive 2DES spectra, this leads to a dispersive lineshape as the blue‐shifted ESA appears with negative sign (ΔR/R<0), in contrast to the partially overlapping ground state bleaching (GSB) and stimulated emission (SE) signals (ΔR/R>0). The optical properties of the J‐aggregated excitons are therefore described as those of an effective three‐level system comprising ground state, (one‐) exciton, and blue‐shifted two‐exciton states [49]. For the squaraine dyes used in the present manuscript, we measured a ∼3 meV blue‐shift of the two‐exciton state for an aggregate formed on a planar gold surface [22]. We have carefully studied the pump fluence dependence of the nonlinear response of the investigated sample and see no significant effect on the line shape when increasing the pump fluence by a factor of 3 over that used in Figure 2 (10 μJ/cm^2^). This supports that the two‐exciton blueshift is indeed the dominant nonlinearity and that other effects, such as pump‐induced changes in normal mode splitting (“Rabi contraction”), play a minor role.

**FIGURE 2:**
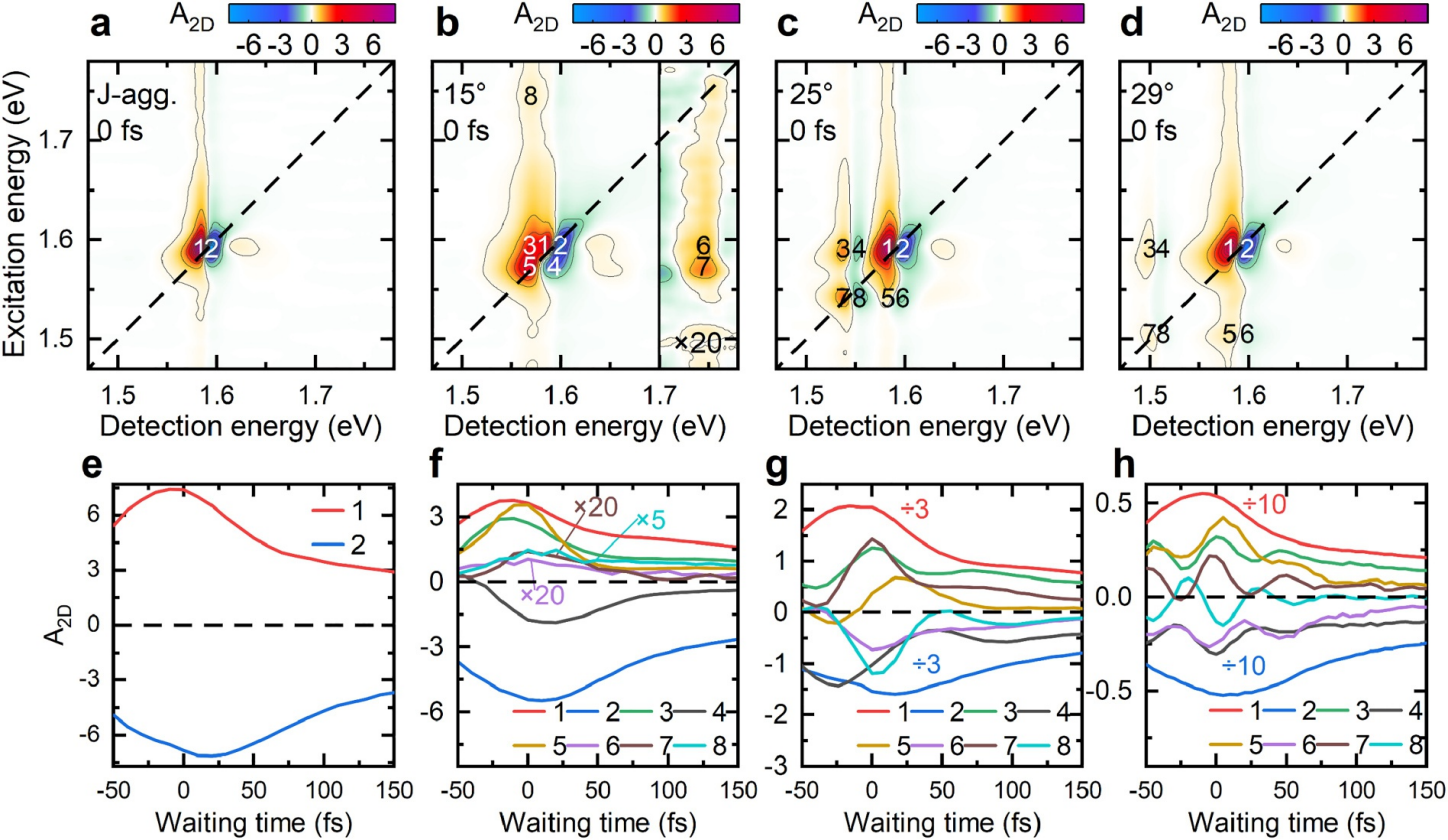
2DES maps and ultrafast peak dynamics of the J‐aggregated thin film and the J‐aggregate/plasmon system. (a) The 2DES map of the J‐aggregated thin film at T=0 reveals a single diagonal peak at the exciton resonance. Its dispersive lineshape is due to the blue‐shift of two‐exciton transitions. (b–d) 2DES maps of the hybrid system at T=0 recorded at incidence angles of θ=15° (b), 25° (c) and 29° (d). The maps reveal diagonal and cross peaks at the LP, X and UP resonances with UP‐LP splittings that can be tuned by varying θ. Distinct UP‐related peaks are only resolved at θ=15°. (e) Waiting time dynamics of selected peaks reflecting the bare exciton dynamics in the J‐aggregated thin film. (f–h) Waiting time dynamics of selected peaks in the 2DES maps of the hybrid system. Oscillations at positive waiting times are distinct signatures of coherent exciton–plasmon couplings with oscillation periods that can be tuned by varying θ. All waiting time dynamics are averaged over a 5 meV × 5 meV energy window.

In the hybrid J‐aggregate/plasmon sample, the 2DES spectrum splits up into a multitude of peaks with dispersive lineshapes. The amplitudes of their nonlinearities are comparable to that of the bare J‐aggregate film. The dependence of these peaks on the incidence angle is seen in Figure 2b–d, depicting 2DES spectra at T=0 fs for three different angles of θ=15°, 25°, and 29°. The angle‐variation of the peak energies is readily understood by comparing them to the linear reflectivity spectra in Figure 1c. All spectra show a peak near the “uncoupled” X resonance around 1.58 eV. LP resonances with dispersive lineshapes are clearly shifted to lower energies along the diagonal. The energy difference between X and LP increases with θ, as already seen in the linear reflectivity (see Figure 1c). Pronounced cross peaks between both resonances are resolved, again with dispersive lineshapes, at all angles. The cross peaks appear both above and below the diagonal, a signature that coherent couplings between SPP and “uncoupled” excitons are involved in the polariton formation in the hybrid system [26]. We emphasize that numerical simulations convincingly show (Figure S11 in [4]) that these “uncoupled” excitons are localized in‐between the slits and that their contributions to the spectra are only seen when taking these regions of the sample into account. This suggests that peaks at the uncoupled exciton resonance are not related to pronounced electromagnetic field gradients across the exciton layer. Such gradients might, in principle, result in weaker couplings of excitons in the region of the layer that is more separated from the metal surface.

In the spectra at higher angles (25° and 29°), clear signatures of UP resonances are difficult to resolve and most of the peak structure can be understood in terms of LP and “uncoupled” X resonances. For 15°, this is not the case. Here, signatures of UP are observed along the diagonal (at ∼1.75 eV) and in the form of cross peaks with LP and X above and below the diagonal. The (X,UP) cross peaks are another indication that the “uncoupled” X play a central role for the polariton formation. The presence of such peaks is unexpected based on simplified two‐state models that are often used to interpret the signatures of X‐SPP coupling in polaritonic systems [27, 42]. Such simplified models evidently predict cross‐peaks between UP and LP only. In our spectra such peaks are present, yet their amplitude is weak. We will discuss below that this is a result of the interplay between the different transition dipole moments of UP and LP and the angle‐dependent exciton fraction in the polariton resonances.

For drawing such conclusions, it is especially instructive to analyze the waiting time evolution of the 2DES peaks. In the bare J‐aggregate thin film (Figure 2e), the amplitudes of the maxima and minima of the dispersive X peak show a rapid partial decay, reducing in amplitude by more than a factor of 2 within the first ∼100 fs [24, 51]. This is a characteristic signature of the initial exciton dynamics in the disordered J‐aggregate, in which coupling to molecular vibrations results in a rapid relaxation of the optically induced exciton distribution [51, 52].

Additionally, the dynamics of all polariton peaks in the hybrid sample show a rapid amplitude decay during the first 100 fs. Here, additional relaxation processes, specifically the radiative damping of the plasmonic system and their Ohmic losses become important [53, 54]. Importantly, the time resolution of our setup is sufficiently high to probe the coherent dynamics of the polaritonic peaks before they are destroyed by such ultrafast decoherence processes [5]. We observe clear oscillatory modulations in the amplitudes of essentially all polariton peaks. For θ=15°, the oscillation period is so long (∼200 fs) that they are difficult to resolve. For θ=25°, the dominant oscillation period is ∼85 fs, in good agreement with the X‐LP splitting at this angle of 45 meV. For the largest angle of 29°, the oscillation period decreases to ∼50 fs, again agreeing well with the X‐LP splitting of 85 meV. A detailed analysis of the oscillation periods observed at several additional angles is reported in Figure 3 of Ref. [4]. Importantly, such oscillations of the peak amplitudes are seen not only for positive T but also for negative waiting times. For T>0, the time evolution of the 2DES map probes population dynamics, whereas for T<0 the measured signals reflect a pump‐induced perturbed free induction decay [55, 56]. It is evident from the data that the energetic splitting between X and LP governs the oscillation period. Oscillations with a period corresponding to the UP–LP splitting are difficult to resolve in this structure. Evidence for such UP–LP oscillations is only found close to the crossing angle. This information cannot be deduced from angle‐resolved linear spectroscopy in which “uncoupled” excitons appear as an angle‐independent resonance. Therefore, the linear spectra alone give no insight into the role of these “uncoupled” X for the polariton formation.

**FIGURE 3:**
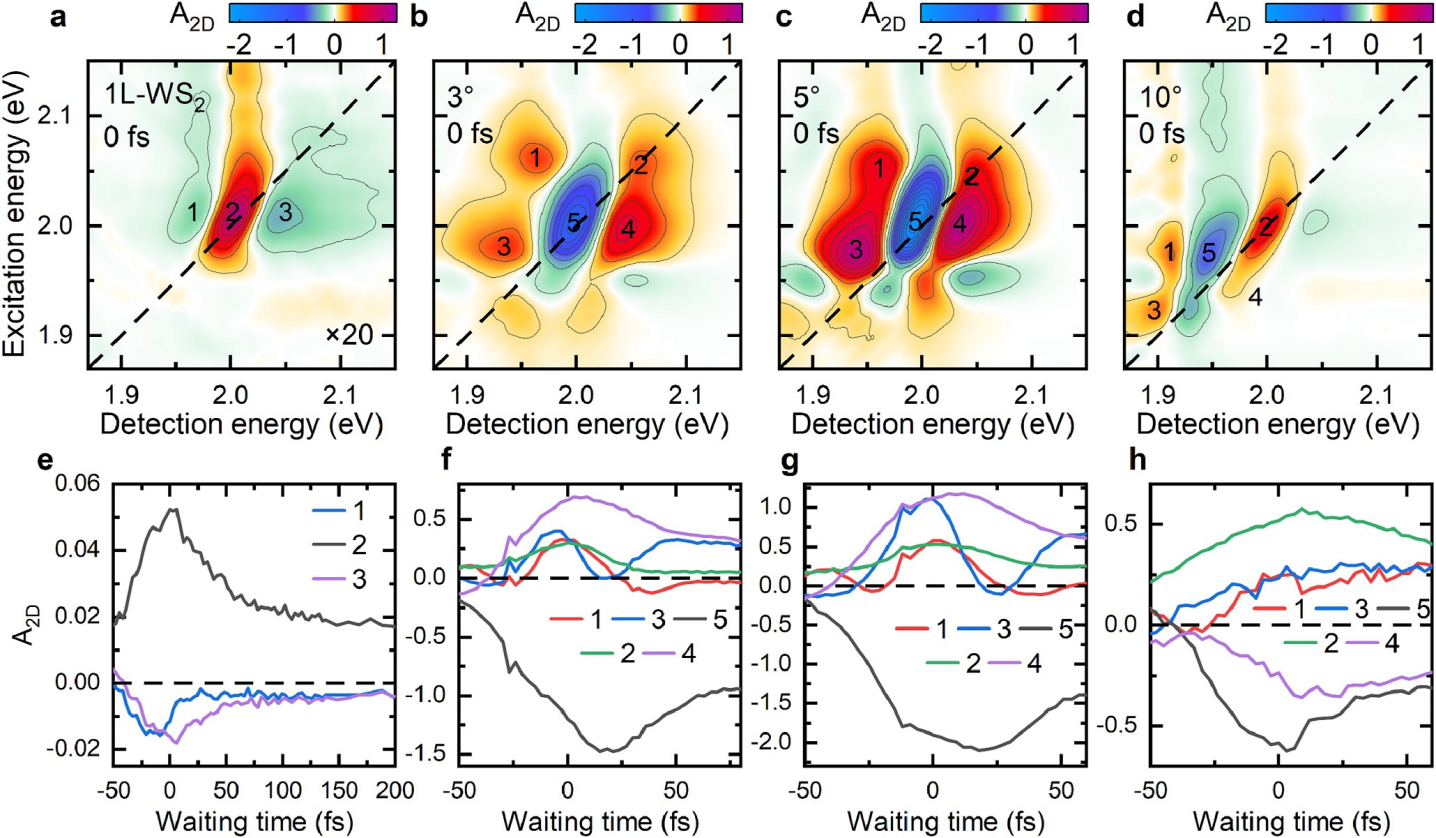
2DES maps and ultrafast peak dynamics of the WS_2_ monolayer and the 1L‐WS_2_/plasmon hybrid system. (a) The 2DES map of 1L‐WS_2_ at T=0 reveals a weak A‐exciton nonlinearity that mainly results from an excitation induced dephasing of the exciton resonance. The data was measured with 30 μJ/cm^2^ pump fluence and scaled by 1/3 for direct comparability with the data presented in (b–d). (b–d) 2DES maps of the hybrid system at T=0 recorded at incidence angles of θ=3° (b), 5° (c) and 10° (d). Compared to 1L‐WS_2_, the nonlinearity is enhanced by more than an order of magnitude and changes sign at the exciton resonance. A sequence of positive and negative peaks reflects the nonlinear response of UP and LP resonances. Data for the hybrid sample were measured with 10 μJ/cm^2^ pump fluence. (e) Waiting time dynamics of selected peaks reflecting the bare exciton dynamics in the 1L‐WS_2_ layer. (f–h) Waiting time dynamics of selected peaks in the 2DES maps of the hybrid system. Oscillations at positive waiting times are signatures of the coherent response of the polariton system. All waiting time dynamics are averaged over a 5 meV × 5 meV energy window.

Before turning to the hybrid 1L‐WS_2_/plasmon sample, we briefly discuss the salient features of 2DES maps of a bare 1L‐WS_2_ sample, as measured on a planar Al_2_O_3_‐coated silver substrate. Figure 3a shows the 2DES spectrum for T=0 fs in the spectral region of the A‐exciton resonance at ∼2 eV. The lineshape of the exciton peak on the diagonal shows a central positive peak with two asymmetric negative sidelobes along the detection energy. This is a characteristic lineshape for semiconductor excitons for which the nonlinearity is dominated by many‐body interactions [26, 40, 41, 57, 58, 59, 60]. Very qualitatively, such many‐body interactions can affect the excitonic resonance in three ways. They can reduce the oscillator strength via phase–space filling, resulting in a (positive) bleaching nonlinearity. Excitation‐induced shifts (EIS) of the exciton resonance manifest as a dispersive lineshape in ΔR/R, similar to the two‐exciton blue‐shift in the J‐aggregated thin film. Lastly, excitation‐induced dephasing (EID), that is, an increase in the dephasing rate due to exciton–exciton interactions, results in a broadening of the exciton resonance, showing up as a positive peak with negative sidelobes in the nonlinearity [61]. The 2DES map in Figure 3a shows such an EID lineshape. A slight asymmetry of the lineshape along the detection energy axis indicates finite contributions of EIS. For 1L‐TMDs, it has been shown that EID indeed is the dominant nonlinearity, whereas phase–space filling and EIS were found to be weaker [5, 58, 60, 62]. The tilt of the 2DES peak along the diagonal is an indication of a finite inhomogeneous broadening, likely reflecting local strain and disorder in the monolayer [62, 63].

For the 1L‐WS_2_/plasmon hybrid sample, 2DES maps are shown in Figure 3b–d for T=0 fs at three selected incidence angles θ=3° (Δ>0, Figure 3b), θ=5° (Δ≈0, Figure 3c), and θ=10° (Δ<0, Figure 3d). Two aspects of the maps are particularly striking. First, the nonlinearity of the hybrid sample is more than an order of magnitude stronger than that of the bare monolayer under comparable excitation conditions. Second, the lineshape of 2DES maps of the hybrid sample differs significantly from that of 1L‐WS_2_. In particular for small incidence angles, the sign of $A_{2D}$ along $E_{\det}$ is effectively reversed. We will show that this enhancement is so large because of the very different oscillator strengths of the TMD excitons and the SPP modes. Essentially, the polaritons inherit a large oscillator strength from the SPP, as seen in Figure 1e, whereas their optical nonlinearity is determined by the TMD excitons. It is therefore the interplay between excitonic and plasmonic fraction that lies at the heart of the enhanced polariton nonlinearity. We observe the strongest enhancement close to the crossing. Hybridization with the SPP also results in a pronounced modification of the 2DES lineshape. In particular for θ=3° and θ=5°, a strong ESA contribution is seen on the diagonal, close to $E_{X}$ (peak 5). Four additional positive peaks (peaks 1–4) show up as main features, located at lower and higher detection energies and close to the UP and LP energies along the excitation. We have recently shown [5] and will argue below that this rather complex lineshape results from the spectral overlap of dispersive and absorptive polaritonic diagonal and cross‐peaks. Specifically, all 2DES spectra feature dispersive diagonal peaks at both LP and UP resonances. The negative parts of these diagonal polariton peaks merge into the central negative peak (5) while their positive parts are individually resolved as peaks 2 (UP) and 3 (LP). In contrast, the cross‐peak shapes change as a function of detuning Δ [31]. For small detunings (θ=3° and θ=5°) they have an essentially positive, absorptive shape. The above‐diagonal ($E_{\mathrm{ex}}=\text{UP},E_{\det}=\text{LP}$) cross‐peak (labeled peak 1) in Figure 3b,c appears at the LP energy. This energy marks the zero‐crossing of the dispersive (LP,LP) diagonal peak. Equally, the (LP,UP) cross‐peak below the diagonal appears at the detection energy of the UP resonance, marked as peak 4. Because our sample is at the border of strong coupling, these peaks overlap partially. In the case of considerable detuning (θ=10°, Figure 3d), clear changes in the 2DES lineshape are seen. The lineshape of (UP,UP) now approaches that of the uncoupled A‐exciton in Figure 3a while the lineshape of (LP,LP) remains dispersive. The (UP,LP) cross‐peak now shows a similar dispersive lineshape as the (LP,LP) peak. The (LP,UP) cross‐peak essentially vanishes. For such negative detuning, UP becomes more exciton‐like while LP becomes more SPP‐like [44]. As such, the UP nonlinearity approaches that of the “uncoupled” X while the LP nonlinearity will vanish since its large SPP fraction suppresses the nonlinearity [5].

The waiting time dynamics for the bare 1L‐WS_2_ sample are shown in Figure 3e. They show a partial 30‐fs decay that can be rationalized in terms of an optical–phonon‐induced relaxation into momentum–dark exciton states. Different dynamics of the negative side lobes arise from a time‐dependent EIS contribution [5, 60]. The dynamics for peaks 1‐5 of the hybrid 1L‐WS_2_/plasmon sample are shown in Figure 3f–h.

For small detunings (Figure 3f,g), substantial oscillations of the peak amplitudes are seen within the first 50 fs that reflect the dynamics of coherent polariton excitations during the dephasing time [5]. For the largest detuning in our study, θ= 10° (Figure 3h), these oscillations are largely suppressed. In contrast to the J‐aggregate/plasmon hybrid sample, the polariton linewidths of the 1L‐WS_2_/plasmon sample are much broader. This makes the corresponding polariton dephasing so rapid that multiple UP–LP Rabi oscillations are no longer resolved. Beyond this typical dephasing time of 50 fs, no further changes of the 2DES lineshapes are observed and the polariton nonlinearity decays slowly on a picosecond timescale [5]. Such long‐lived polariton nonlinearities cannot be explained based on commonly employed two‐state X‐SPP models, which predict a rapid polariton decay, on a 100fs timescale, through radiative and/or ohmic losses [5]. We rationalize these rather long polariton lifetimes by considering polariton relaxation into dark exciton states in our sample.

The dynamics of the peak amplitudes and the evolution of the 2DES lineshapes during the first 100 fs provide significant insight into the system dynamics. To demonstrate this, we show 2DES maps at selected waiting times for both hybrid samples for small detunings, that is, θ=25° for the J‐aggregate/plasmon sample (Figure 4a–d) and θ=5° for the 1L‐WS_2_/plasmon sample (Figure 4e–h). For the J‐aggregate/plasmon sample, the nonlinearity reduces quickly, most prominently for the LP‐associated peaks. The Rabi oscillation dynamics seen in Figure 2g can best be discerned in the ESA contributions. After the dephasing time, the only change seen in the map is a “straightening” of the individual 2DES peaks, that is, a loss of excitation energy dependence of their lineshape. Even after the dephasing time, all peaks retain their dispersive lineshapes.

**FIGURE 4:**
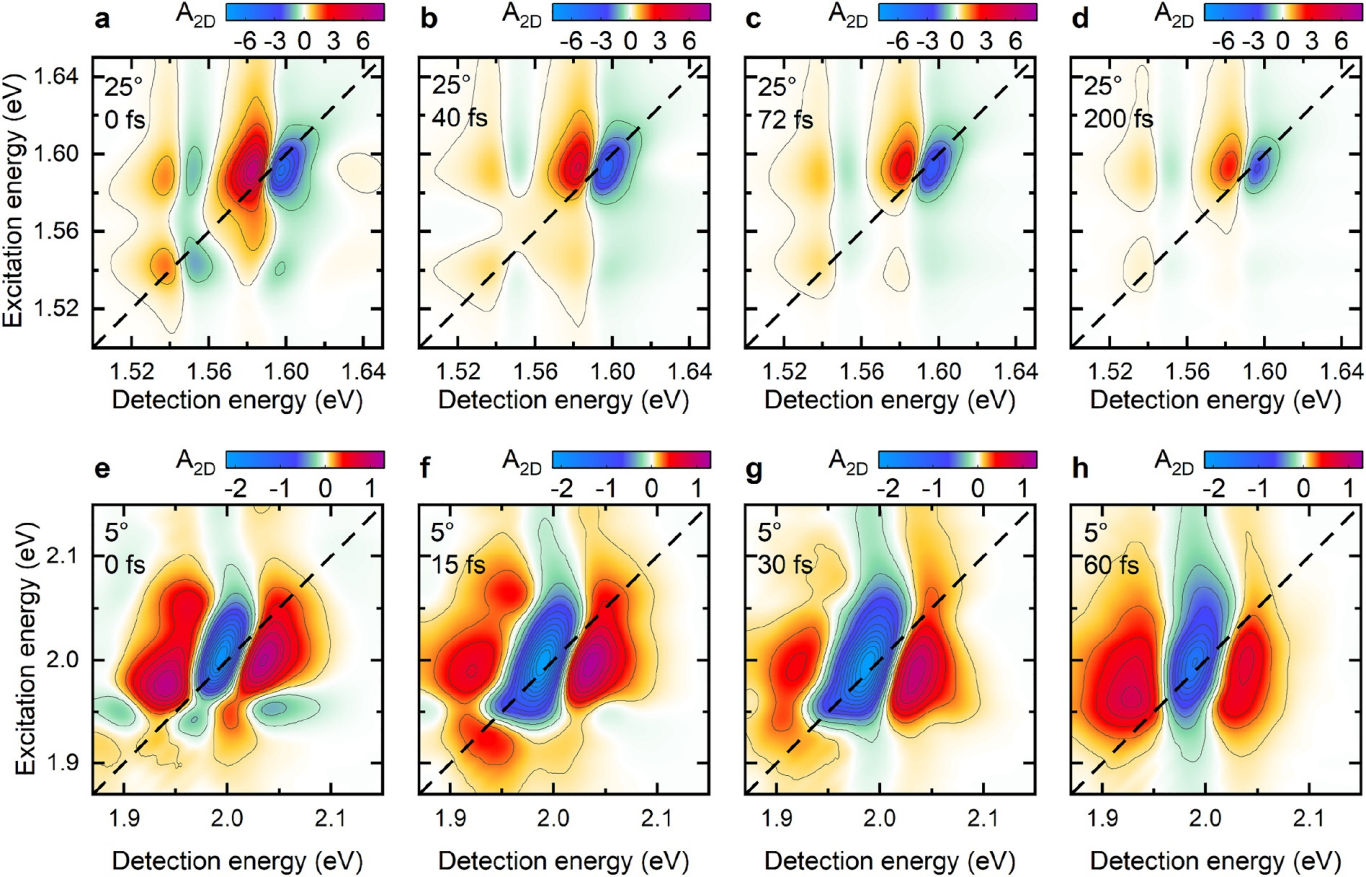
Temporal evolution of the 2DES lineshape of the hybrid exciton/plasmon nanostructures. (a–d) 2DES map of the J‐aggregate/plasmon hybrid recorded for an angle of incidence of *θ* = 25° and increasing waiting times T. Although, at early times, the 2DES maps shows pronounced signatures of coherent polariton excitations, the response after 200 fs is mainly induced by an incoherent population of J‐aggregate excitons. The time evolution marks transition from coherent polariton to incoherent exciton response. (e–h) 2DES map of the 1L‐WS2/plasmon hybrid recorded for an angle of incidence of θ=5° and increasing waiting times T. Also in this system, the shape of the 2DES maps evolves significantly during the first 60 fs. This marks the transition from a coherent polariton response to an optical response that is mainly induced by a finite population of optically dark exciton states.

For the 1L‐WS_2_/plasmon sample (Figure 4e–h), we observe a much reduced drop in nonlinearity. During the dephasing time, that is, for waiting times < 50 fs, strong modulations of the 2DES spectra reflect the oscillatory peak dynamics of the coherent polariton excitations that have been exemplified in Figure 3g. After this coherent polariton regime (Figure 4h), the 2DES map has changed significantly compared to T=0 fs (Figure 4e). Whereas the map at early times is characterized by dispersive diagonal and positive cross‐peaks, the 2DES maps at later times only show dispersive diagonal and cross‐peaks. As in the J‐aggregate/plasmon sample, this reflects the loss of excitation energy dependence in the 2DES maps that is caused by the rapid loss of polariton coherence in the sample. The observed qualitative changes in the polariton cross‐peak lineshape for the TMD‐based hybrid strongly contrast the observations for the J‐aggregate‐based sample which does not show such a change in polariton lineshape.

We rationalize the 2DES spectra of both hybrid exciton/plasmon samples using effective minimal coupling Hamiltonians. Both samples comprise a nanoslit array with subwavelength period [4, 27, 53, 64]. Such a plasmonic grating supports strongly localized and spatially highly structured optical near‐fields. For p‐polarized excitation, calculations of the optical near‐field profiles [4, 5] suggest a local field enhancement of their in‐plane component of ∼10 while the in‐plane fields outside the slits are at least a factor of five weaker.

We consider the optical excitations of the J‐aggregated thin film as localized Frenkel‐type excitons [37, 50, 65]. Near‐field dipole‐dipole coupling between neighboring molecules of the aggregate results in a delocalization of the electronic molecular wavefunctions, forming superradiant J‐aggregated excitons which are delocalized over typically 10–25 molecular units [37, 65, 66]. This delocalization length is limited by an interplay between disorder‐induced variations of the molecular energy levels and phase breaking scattering events, for example, due to electronic couplings to nuclear vibrations. Hence, J‐aggregate excitons are localized on a deep subwavelength scale, much smaller than the typical dimensions of the optical near fields in the plasmonic grating. This exciton localization has two important consequences. Firstly, their coupling to the optical near‐fields can effectively be described in point–dipole approximation. This implies that, qualitatively, two different excitonic species can be identified: Strongly coupled excitons ($X_{S}$) located inside the slits of the nanoslit array and weakly coupled excitons ($X_{W}$) in the regions in‐between the slits. Both types of excitons will show different effective coupling strengths ($V_{S}$ and $V_{W}$) to the plasmonic mode (|P〉) (Figure 5a). Second, the fermionic nature of their molecular excitations results in a characteristic blue‐shift of their two‐exciton states [22, 49] (Figure 5a). This constitutes the dominant optical nonlinearity of the localized J‐aggregate excitons. Other many‐body effects, in particular excitation‐induced dephasing and line shifts that are characteristic for inorganic semiconductors supporting Wannier‐type excitons, are less significant. In contrast to the J‐aggregate excitons, the plasmonic mode has a negligible nonlinearity under our experimental conditions. Therefore, the plasmonic mode can reasonably well be described as a harmonic oscillator. In the two‐quantum manifold (2Q), couplings arise that represent the deexcitation of either a two‐exciton or a mixed exciton‐plasmon (X,P) state and the excitation of the corresponding mixed (X,P) or two‐plasmon (2P) state.

**FIGURE 5:**
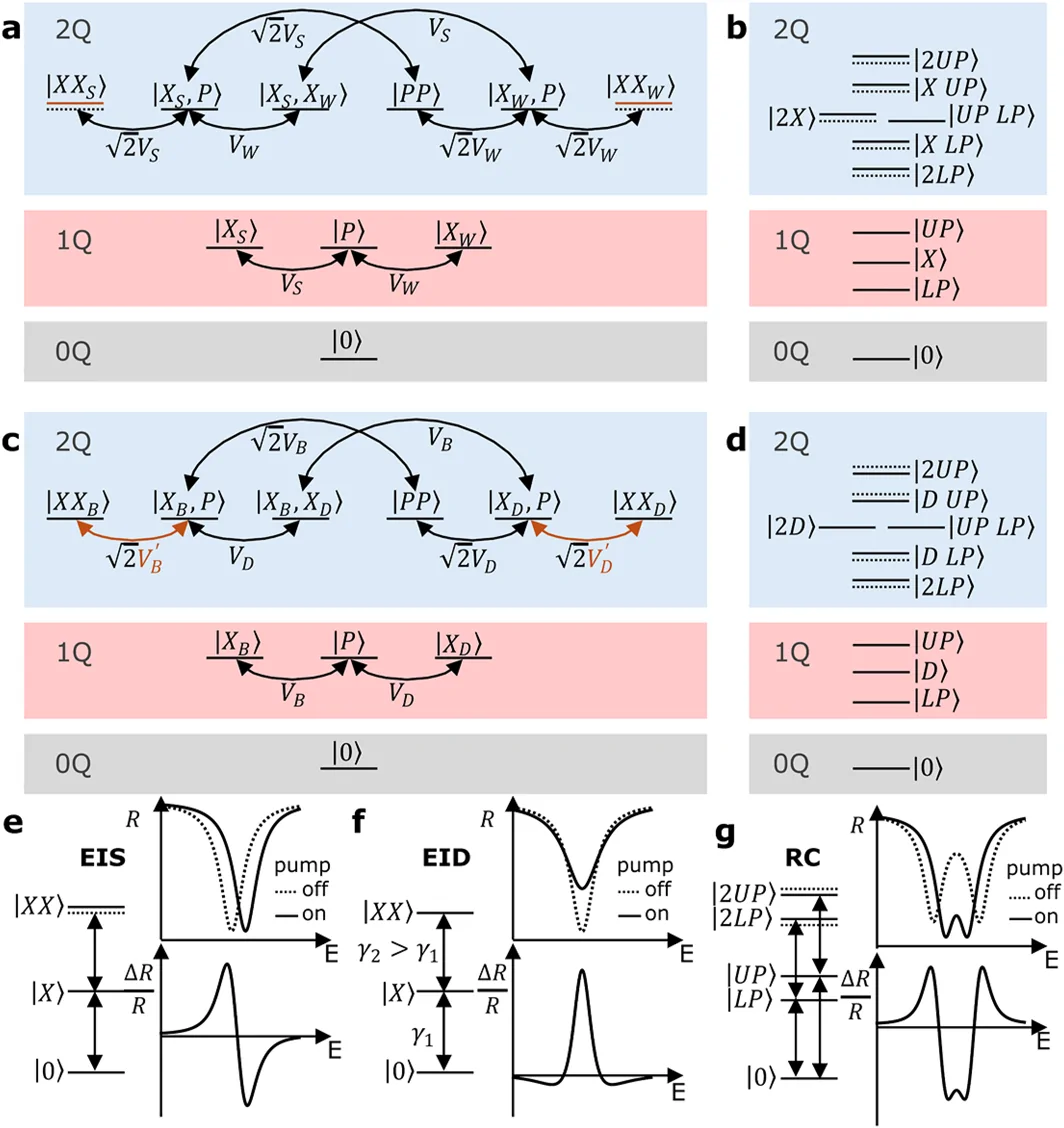
Phenomenological, effective Hamiltonian and origin of the 2DES lineshapes in hybrid exciton/plasmon nanostructures. (a) For the J‐aggregate/plasmon hybrid we consider couplings of the plasmonic mode (|P〉) to ensembles of strongly ($|X_{S}\rangle$) and weakly coupled excitons ($|X_{W}\rangle$). The dominant nonlinearity is a blue‐shift to the two‐exciton states $|XX_{S}\rangle$ and $|XX_{W}\rangle$. (b) This results in blue‐shifts of all two‐quantum polariton states. (c) For the 1L‐WS_2_/plasmon hybrid, we consider a coupling of the plasmonic mode |P〉 to ensembles of bright ($|X_{B}\rangle$) and momentum‐dark excitons ($|X_{D}\rangle$). The dominant nonlinearities are slight changes in the X‐SPP coupling strength in the two‐quantum manifold together with an increase in the dephasing rate of the two‐exciton states (EID). (d) This results in a reduction of the normal mode splitting between the polariton states in the two‐quantum manifold. (e) Origin of the 2DES lineshapes in the J‐aggregated thin film. A blue‐shift of the two‐exciton states (EIS) results in a dispersive lineshape of the differential reflectivity ΔR/R. (f) In 1L‐WS2, EID gives rise to a peak with negative side lobes in ΔR/R. (g) Schematic of a polariton nonlinearity induced by a reduction in X‐SPP coupling in the two‐quantum manifold. The reduction in normal mode splitting (Rabi contraction, RC) induces dispersive lineshapes of the polariton peaks in ΔR/R.

Diagonalization of the Hamiltonian gives the resulting polariton excitations (Figure 5b). In the 1Q manifold, UP and LP appear together with a state |X〉 at the exciton energy. All three excitations appear in the linear optical spectrum of the sample (Figure 1) which, however, cannot reveal how these polaritons emerge from the hybridization of excitons and plasmons. Such information can be retrieved from 2DES [67, 68]. These 2DES spectra not only probe optical transitions to the 1Q states but also ESA from the 1Q to the 2Q manifold. In the polariton basis, these can be grouped into double excitations of UP and LP, a mixed |UPLP〉 state, doubly excited *X* and mixed |XUP〉 and |XLP〉 states. Because of Pauli blocking, all those 2Q states that contain a blue‐shifted two‐exciton state as their constituent will show a blue‐shift with respect to a harmonic oscillator model. Therefore only |UPLP〉 appears unshifted. Hence, the two‐exciton blue‐shift also emerges as the dominant polariton nonlinearity. It manifests itself in predominantly dispersive lineshapes of all polariton peaks in the 2DES maps (Figure 2) with the same symmetry as for the J‐aggregates excitons (Figure 5e).

Simulations of the 2DES maps and their waiting time dynamics based on such an effective Hamiltonian for the J‐aggregate/plasmon hybrid sample have been presented in Ref. [4]. The simulation can convincingly describe the experimental results in Figures 2 and 4. Our sample is characterized by a total effective dipole moment of the collective excitonic modes ($|X_{S}\rangle$ and $|X_{W}\rangle$) that is similar to that of the plasmonic mode. This case can lead to a |UP〉 that is essentially dark near the crossing while exciton–plasmon hybridization results in bright |LP〉 and |X〉 states. In such a scenario, the 2DES maps in the coherent polariton regime are dominated by |LP〉 and |X〉 cross‐ and diagonal peaks that oscillate at the frequency given by their energy difference, as observed experimentally. These oscillations reflect a coherent population transfer between strongly coupled and weakly coupled excitons driven by the plasmon mode. This implies that the plasmonic field induces a non‐local transport of energy between excitons that are localized in the slit regions and excitons outside the slits [4]. This non‐local transport persists during the coherence of the polariton excitations, in our sample approximately 50 fs. In the J‐aggregate/plasmon hybrid, polariton coherence is lost via an interplay of different relaxation processes, including the coupling of excitons to molecular vibrations [52, 69] and the radiative and Ohmic damping of the plasmonic mode [54]. The change in lineshape of the 2DES peaks during the first 100 fs from peaks that are oriented toward the diagonal into peak shapes that are oriented vertically is a signature that vibronic relaxation within the inhomogeneously broadened exciton manifold plays a dominant role for the loss of polariton coherence. This results in incoherent exciton (|X〉) and lower polariton populations. These populations dominate the polariton nonlinearities for waiting times beyond 100 fs. Specifically, the incoherent exciton population gives rise to persistent (X,X) diagonal and (X,LP) cross‐peaks as seen in Figure 4d.

TMD monolayers are characterized by large exciton binding energies in the range of several 100 meV and small exciton Bohr radii in the range of 1–2 nm. For high‐quality samples, effects of disorder and the resulting localization of the excitonic center of mass wave function play substantially less significant roles than for superradiant J‐aggregate excitons. Experimentally, this results in fundamentally different lineshapes of the nonlinear optical response of J‐aggregated thin films and TMD monolayers. In contrast to the dispersive J‐aggregate lineshape, TMD monolayers show a lineshape that is characteristic for an excitation‐induced dephasing of the excitonic polarization [40, 58, 60]. The significant extent of their center‐of‐mass wavefunction within the TMD plane makes the excitonic polarization susceptible to interactions with neighboring excitons.

The effect of such interactions on their optical properties is often described in terms of optical Bloch equations for the interband polarization, modified by the Coulomb interactions among the electrons [70]. For the case of strongly Coulomb‐bound TMD excitons, these equations have been reformulated in terms of the TMD Bloch equations to include valley‐resolved dispersion relations. The resulting equation of motion for the exciton amplitude in the coherent limit has been discussed for instance in Ref. [57] and used to simulate linear and nonlinear optical properties of TMD monolayers. In the vicinity of plasmonic nanostructures, the strong spatial confinement of the plasmonic fields results in a coupling to both momentum‐bright excitons, accessible by far field light, and to momentum–dark excitons with in‐plane momenta beyond the light cone [2]. As a consequence, a minimal model of three coupled oscillators has been proposed for plasmon/TMD hybrid structures [1]. In this model, momentum–bright $|X_{B}\rangle$ and nearly‐degenerate momentum–dark $|X_{D}\rangle$ excitons interact via their mutual coupling to the plasmonic mode |P〉. Here, we translate this into a model Hamiltonian and adopt it to describe the 1Q states of our TMD‐plasmon hybrid (Figure 5c). This, again, results in three 1Q states in the polariton basis (Figure 5d). This suggests the possibility for a three‐peak structure in the linear optical spectra in agreement with experimental observations for TMD‐based hybrids [66]. In our system, however, the effective transition dipole moment of the monolayer excitons is much weaker than that of the plasmonic mode. As a result, UP and LP appear with an oscillator strength that is much larger than that of $|X_{B}\rangle$ and $|X_{D}\rangle$. Also the polaritonic |D〉 state near the exciton energy has a much weaker oscillator strength than UP and LP. It is therefore justified to consider |D〉 as an effective dark state.

To model the nonlinear spectra, we extend this Hamiltonian by including the 2Q manifold of the coupled harmonic oscillators. 2DES spectra simulated based on this Hamiltonian and their waiting time dynamics are presented in Ref. [5] and are in reasonable agreement with experiment. In these simulations, we have included the 2Q manifold of the coupled harmonic oscillators. As in the case of the J‐aggregate/plasmon hybrid, this gives rise to analogous 2Q states, except for the relabeling of the excitonic 1Q states. Additionally, in the polariton basis, X is relabeled as D to symbolize its dark nature.

An optical nonlinearity of the TMD/plasmon hybrid, that is, a deviation from a harmonic oscillator model, is phenomenologically introduced in two ways: (i) A substantial body of experimental [40, 60, 62] and theoretical [58] studies over the past years have shown that an excitation‐induced dephasing of the exciton polarization is indeed the dominant nonlinearity of a bare TMD monolayer at room temperature. This is included by adding an additional dephasing term for the one‐exciton to two‐exciton transition [60, 62]. (ii) A second and physically different origin of the nonlinearity may appear in the hybrid exciton–plasmon system. In this case, the normal mode splitting results from the collective coupling of the TMD excitons to the plasmonic mode. It therefore scales with the excitonic optical polarization that is created by the coupling to the plasmonic field. The optical excitation of the hybrid system by the pump laser may reduce this polarization by creating two‐exciton polarizations [58]. This “Pauli blocking” quenching of the exciton–light interaction will effectively reduce the normal mode splitting. This optical nonlinearity, sometimes termed “Rabi contraction” [71], is considered by assuming that phase–space filling of excitonic states upon excitation will result in a slight reduction of the transition dipole moment for the one‐exciton to two‐exciton transitions. This also reduces the coupling matrix elements in the 2Q manifold that are marked in red in Figure 5c. The consequence of this change in transition dipole moment is a slight reduction of the NMSs in the 2Q states in the polariton basis. This is illustrated in Figure 5d as a shift between the dashed lines (2Q states in the absence of Pauli blocking) and the solid lines (with Pauli blocking). In molecular polariton systems, the resulting energy shifts are referred to as Rabi contractions [3, 72, 73]. These two nonlinearities have different effects on the lineshape of the 2DES maps. EID, that is, a broadening of the ESA, results in a characteristic EID lineshape that is schematically depicted in Figure 5f. In a polaritonic system the effect of Pauli blocking (“Rabi contraction”) is quite different. As in the J‐aggregate, it results in dispersive polariton peaks in the 2DES maps but now with different symmetry for UP and LP (Figure 5g). We expect a negative ΔR/R signal in‐between the two polaritons, opposite in sign to a typical exciton bleaching or EID nonlinearity.

The signatures of such a Pauli blocking/Rabi contraction nonlinearity are most prominently seen in Figure 3b,c, for waiting times around zero. They persist during the coherence time of the polaritonic system (Figure 4e–g) for waiting times of up to ∼40 fs. This manifestation of this coherent polariton response in the 2DES maps is distinctly different from an incoherent response seen for later waiting times (Figure 4h). Relaxation within the polariton manifold results in a rapid population transfer into dark exciton states (|D〉). These dark excitons will also contribute to a Rabi contraction. Yet, the resulting 2DES maps no longer show the distinct correlation between excitation and detection energies that are seen at earlier waiting times. Instead, the 2DES map evolves into a vertically‐oriented stripe‐like pattern (Figure 4h). This is the characteristic marker for an incoherent polariton nonlinearity in the 2DES maps.

## 3 Summary and Outlook

We have reported a comparative study of ultrafast angle‐resolved 2DES spectra with 10‐fs time resolution of two prototypical strongly coupled exciton/plasmon hybrids. Both comprise a metallic nanoslit array as a plasmonic nanoresonator, covered with a thin layer of a J‐aggregate of squaraine molecules or an atomically thin semiconductor, and a monolayer of tungsten disulfide. In both systems, we resolve the transition from a coherent polariton nonlinearity, marked by a coherent energy transfer between excitons and plasmons, toward an incoherent nonlinearity governed by dark exciton populations. The transition occurs on a 50‐fs time scale, largely set by a rapid relaxation within the polariton manifold and into dark states that is driven by the coupling to vibrational modes of the excitonic layer.

The microscopic optical nonlinearities that are observed in the two systems are qualitatively different. In the J‐aggregate/plasmon hybrid, the nonlinearity is induced by a characteristic blue‐shift of the doubly‐excited Frenkel exciton states. For these disordered aggregate systems, with center‐of‐mass wave functions of their Frenkel excitons that are localized on a few‐nm length scale, the optical pump has little effect on the normal mode splitting. This is very different for the TMD/plasmon hybrid. Here, disorder effects play a much smaller role, and many‐body interactions significantly impact their nonlinear response. Our results show that the nonlinearity of the TMD/plasmon hybrid mostly arises from a Pauli blocking nonlinearity of the excitonic system, with weaker contributions from other many‐body interactions, predominantly excitation‐induced dephasing. The second characteristic difference between the two systems is the very different oscillator strengths of their excitonic constituents. The large oscillator strength of the J‐aggregate layer results in practically dark upper polariton modes while lower polaritons and polaritonic transitions near the exciton resonance appear with comparable strength. This leads to a dynamic evolution of the 2DES maps reflecting a plasmon‐mediated coherent population transfer of excitons that are localized in regions of the sample with different amplitude of the plasmonic near field.

In the TMD‐based hybrid, the exciton absorption, and therefore the optical nonlinearity of the monolayer, is inherently weak. Coupling to the plasmonic system largely enhances the nonlinear optical response by more than an order of magnitude. The origin of this effect appears to be a Pauli blocking of the excitonic states which results in a transient reduction in normal mode splitting of the polaritons. The characteristic lineshape of the 2DES maps monitors this coherent polaritonic response and its transition into an incoherent polariton nonlinearity that is largely driven by a rapid phonon‐mediated relaxation into long‐lived dark exciton states in the system. Comparison to model simulations based on an effective Hamiltonian for the one‐quantum and two‐quantum excitations can qualitatively reproduce the experimental 2DES maps and their time evolution in both systems. Such simulations appear as an important tool for interpreting 2DES maps in dissipative strongly‐coupled hybrid systems.

We trust that this comparative study will be helpful for the future design of strongly‐coupled quantum systems and their applications in ultrafast and efficient all‐optical switching [6] and quantum sensing. Our results specifically suggest that TMD‐based hybrids and metamaterials [29] offer intriguing perspectives for such applications.

## Author Contributions

**Daniel C. Lünemann:** visualization, writing – original draft, validation, investigation, formal analysis, data curation, methodology. **Daniel Timmer:** writing – original draft, investigation, formal analysis, validation, data curation, software, methodology. **Antonietta De Sio:** supervision, validation, funding acquisition, methodology, writing – original draft. **Christoph Lienau:** writing – original draft, supervision, validation, conceptualization, project administration, funding acquisition, resources, methodology.

## Funding

Deutsche Forschungsgemeinschaft (SFB 1372 “Magnetoreception and navigation in vertebrates” (project number 395940726), Li 580/16‐1, DE 3578/3‐1), Niedersächsische Ministerium für Wissenschaft und Kultur (DyNano and Wissenschaftsraum ElLiKo).

## Conflicts of Interest

The authors declare no conflicts of interest.

## Data Availability

The datasets generated and/or analyzed during the current study are available from the corresponding author upon reasonable request.
